# Assessing the clinical and cost-effectiveness of inpatient mental health rehabilitation services provided by the NHS and independent sector (ACER): protocol

**DOI:** 10.1186/s12888-024-05524-6

**Published:** 2024-02-06

**Authors:** Helen Killaspy, Christian Dalton-Locke, Caroline S Clarke, Gerard Leavey, Artemis Igoumenou, Maurice Arbuthnott, Katherine Barrett, Rumana Omar

**Affiliations:** 1 Division of Psychiatry, University College London, 6th Floor, Maple House, 149 Tottenham Court Road, W1T 7NF London, UK; 2 Camden and Islington NHS Foundation Trust, St Pancras Hospital, 4 St Pancras Way, NW1 0PE London, UK; 3 Research Department of Primary Care and Population Health, University College London, UCL Medical School, Upper 3rd Floor, Royal Free Campus, Rowland Hill Street, NW3 2PF London, UK; 4 Bamford Centre for Mental Health & Wellbeing, Ulster University, Cromore Road, Coleraine, County, BT52 1SA Londonderry, UK; 5 Barnet, Enfield and Haringey Mental Health NHS Trust, St. Ann’s Hospital, St Ann’s Rd, N15 3TH London, UK; 6 North London Service User Research Forum, Division of Psychiatry, University College London, 6th Floor, Maple House, 149 Tottenham Court Road, W1T 7NF London, UK; 7 Department of Statistical Science, University College London, 1-19 Torrington Place, WC1E 7HB London, UK

**Keywords:** Mental health, Severe mental illness, Inpatient, Rehabilitation, NHS, Independent sector, Clinical effectiveness, Cost effectiveness, Length of stay, Instrumental variable

## Abstract

**Background:**

Mental health rehabilitation services provide specialist treatment to people with particularly severe and complex problems. In 2018, the Care Quality Commission reported that over half the 4,400 mental health inpatient rehabilitation beds in England were provided by the independent sector. They raised concerns that the length of stay and cost of independent sector care was double that of the NHS and that their services tended to be provided much further from people’s homes. However, there has been no research comparing the two sectors and we therefore do not know if these concerns are justified. The ACER Study (Assessing the Clinical and cost-Effectiveness of inpatient mental health Rehabilitation services provided by the NHS and independent sector) is a national programme of research in England, funded from 2021 to 2026, that aims to investigate differences in inpatient mental health rehabilitation provided by the NHS and independent sector in terms of: patient characteristics; service quality; patient, carer and staff experiences; clinical and cost effectiveness.

**Methods:**

ACER comprises a:1) detailed survey of NHS and independent sector inpatient mental health rehabilitation services across England; 2) qualitative investigation of patient, family, staff and commissioners’ experiences of the two sectors; 3) cohort study comparing clinical outcomes in the two sectors over 18 months; 4) comprehensive national comparison of inpatient service use in the two sectors, using instrumental variable analysis of routinely collected healthcare data over 18 months; 5) health economic evaluation of the relative cost-effectiveness of the two sectors. In Components 3 and 4, our primary outcome is ‘successful rehabilitation’ defined as a) being discharged from the inpatient rehabilitation unit without readmission and b) inpatient service use over the 18 months.

**Discussion:**

The ACER study will deliver the first empirical comparison of the clinical and cost-effectiveness of NHS and independent sector inpatient mental health rehabilitation services.

**Trial registration:**

ISRCTN17381762 retrospectively registered.

## Background

Most people diagnosed with a severe mental illness such as schizophrenia recover at least partially, but around 25% develop longer term, complex problems that require mental health rehabilitation services [1, 2]. These include persistent, severe ‘positive’ symptoms (delusions and hallucinations) and ‘negative’ symptoms (reduced motivation, verbal communication and emotional reactivity), alongside poor organisational skills due to specific cognitive impairments associated with the illness. Recovery is often further complicated by substance misuse and co-morbid physical health problems (such as obesity, diabetes, cardiovascular and pulmonary disease). Some people also have pre-existing conditions such as intellectual impairment and/or developmental disorders, including those on the autism spectrum. These multiple problems impact negatively on the person’s social and everyday functioning (self-care, housework, shopping, cooking, budgeting and interpersonal skills) and may lead to challenging behaviours [3]. Many will struggle to engage in community activities or gain employment, and over half are vulnerable to self-neglect and exploitation [4, 5]. In short, without appropriate treatment and support, quality of life for people with complex psychosis is poor.

Due to their complexity, admissions to inpatient rehabilitation services are longer than general (‘acute’) mental health admissions and community support costs for this group are high; it has been estimated that around half the total health and social care budget for mental health is spent on people with complex psychosis [6]. Nevertheless, our previous research has shown that with access to local NHS rehabilitation services, most people can gain the skills to manage with less support over time, graduating from inpatient care to higher, and then lower, supported accommodation [7, 8]. However, recent years have seen major disinvestment in NHS inpatient mental health rehabilitation services across England and increasing reliance on the independent sector.

In 2018, the Care Quality Commission (CQC) surveyed all providers of inpatient mental health rehabilitation services in England [9]. They reported the cost of inpatient rehabilitation to be over £500 m per year, with the independent sector providing around half the 4,400 beds in the country. They noted that use of the independent sector varied greatly by Clinical Commissioning Group (CCG) and admissions to independent sector rehabilitation units were twice as long as those at NHS units. Although the cost per day was similar, admissions to the independent sector therefore cost twice as much. The CQC also found that patients treated in the independent sector were much further from home than those receiving NHS care and they raised concerns about the social dislocation this caused for patients. They highlighted a lack of evidence-based rehabilitation being provided in many units and questioned whether the current system represented an appropriate use of public funds, stating ‘Too often, these…rehabilitation hospitals are in fact long stay wards that institutionalise patients, rather than a step on the road back to a more independent life in the person’s home community.’

The CQC report [9] attracted considerable negative press [10, 11] and prompted a national initiative by NHS England (‘Getting It Right First Time’– GIRFT) to encourage Clinical Commissioning Groups (CCGs) to invest in local NHS mental health rehabilitation services [12]. The recently published NICE Guideline on mental health rehabilitation modelled the potential cost savings if all inpatient rehabilitation were to be provided by the NHS and estimated this at £52,000 per patient (an estimated total saving of over £100 m per year) [13]. However, this may be overly optimistic since there have been no studies comparing the quality and outcomes of NHS and independent sector providers of rehabilitation. A reversal of the current system risks wasting the expertise gained by the independent sector and major investment may be required to rebuild NHS services. Furthermore, there may be bias in the CQC data since NHS Trusts may be transferring patients with higher complex needs to the independent sector, necessitating longer admissions [14, 15].

The Assessing the Clinical and cost-Effectiveness of inpatient mental health Rehabilitation services provided by the NHS and independent sector (ACER) study will be the first to compare the clinical and cost effectiveness of NHS and independent sector inpatient mental health rehabilitation services. Our findings will be of obvious relevance to those who require these services and their families, and will help inform commissioners and policy makers about the most appropriate use of resources for this complex group.

### Aim and research questions

Our overarching aim is to investigate the clinical and cost-effectiveness of inpatient mental health rehabilitation provided by the NHS and independent sector with the objective of investigating differences between them in terms of: patient characteristics; service quality; patient, carer and staff experiences; clinical effectiveness; and their relative cost effectiveness.

Our specific research questions are:

1. Do sociodemographic and clinical characteristics of patients differ between people receiving inpatient rehabilitation in the NHS and the independent sector?
2. Does service quality differ between inpatient rehabilitation units provided by the NHS and the independent sector?
3. Do the experiences of treatment and care, from the perspectives of patients, informal carers and staff, differ between inpatient rehabilitation services provided by the NHS and the independent sector?
4. Is inpatient rehabilitation clinically more effective at preventing readmission when provided by the independent sector or the NHS, after adjusting for differences between the sectors in terms of patient characteristics and length of stay?
5. Is inpatient rehabilitation more cost effective when provided by the independent sector or the NHS, after adjusting for differences between the sectors in terms of key predictors of costs such as patient characteristics and length of stay?

## Methods

### Design

The ACER study is comprised of five Components:

1. a survey of NHS and independent sector inpatient mental health rehabilitation services across England to compare the quality of services and patient characteristics in the two sectors.
2. a qualitative investigation of patient, family, staff and service commissioners’ experiences and perspectives on inpatient mental health rehabilitation provided by the two sectors.
3. a cohort study comparing successful rehabilitation in the two sectors for the patients recruited in Component 1.
4. a service evaluation retrospectively comparing successful rehabilitation over an 18-month period in the two sectors for all users of inpatient rehabilitation services in England on a specific census date, using instrumental variable analysis of routinely collected healthcare record data.
5. a health economic evaluation calculating the relative cost effectiveness of inpatient rehabilitation services provided by the NHS and the independent sector using data from Components 1, 3 and 4.

### Project management

The sponsor for the ACER study is Camden and Islington NHS Foundation Trust.

#### Project Management Group (PMG)

The PMG includes the Chief Investigator (HK, Consultant Psychiatrist and Professor of Rehabilitation Psychiatry) and Co-Applicants (RO, Professor of Medical Statistics; CC, Principal Research Fellow in Health Economics; GL Professor of Mental Health Service Research; AI, Consultant Psychiatrist and Clinical Senior Lecturer; MA, Expert by Experience; KC, Expert by Experience). The PMG is responsible for overseeing the successful progress and completion of the study. The group meets once every two months.

The PMG reviewed and agreed the protocol prior to submission to the Research Ethics Committee (REC) and Health Research Authority (HRA). They will also agree any necessary substantial amendments during the course of the study and submit these to the REC for approval. All local collaborators will be kept informed of any substantial amendments through their nominated responsible individuals.

#### Project Oversight Committee

The independent Project Oversight Committee is chaired by Professor Tom Craig of King’s College London and includes Professor Stephen Bremner (Statistician at Brighton and Sussex Medical School), a patient and public involvement representative, and the Chief Investigator (Professor Helen Killaspy).

#### The North London Service User Research Forum (SURF)

SURF will be consulted throughout the study at four separate points on aspects of the project including any necessary substantial amendments to the study design, interpretation of findings and dissemination.

#### Expert Reference Group

An Expert Reference Group comprising national leaders in mental health rehabilitation service provision has been convened to provide advice to the PMG through the course of the study. Members include Dr Sri Kalidindi, Clinical Lead, NHS England’s ‘Getting it Right First Time’ programme for mental health rehabilitation, and Dr Raj Mohan, immediate past Chair, Faculty of Rehabilitation and Social Psychiatry, Royal College of Psychiatrists. This group will meet on four occasions during the study.

#### Project management organisation

**Fig. 1:**
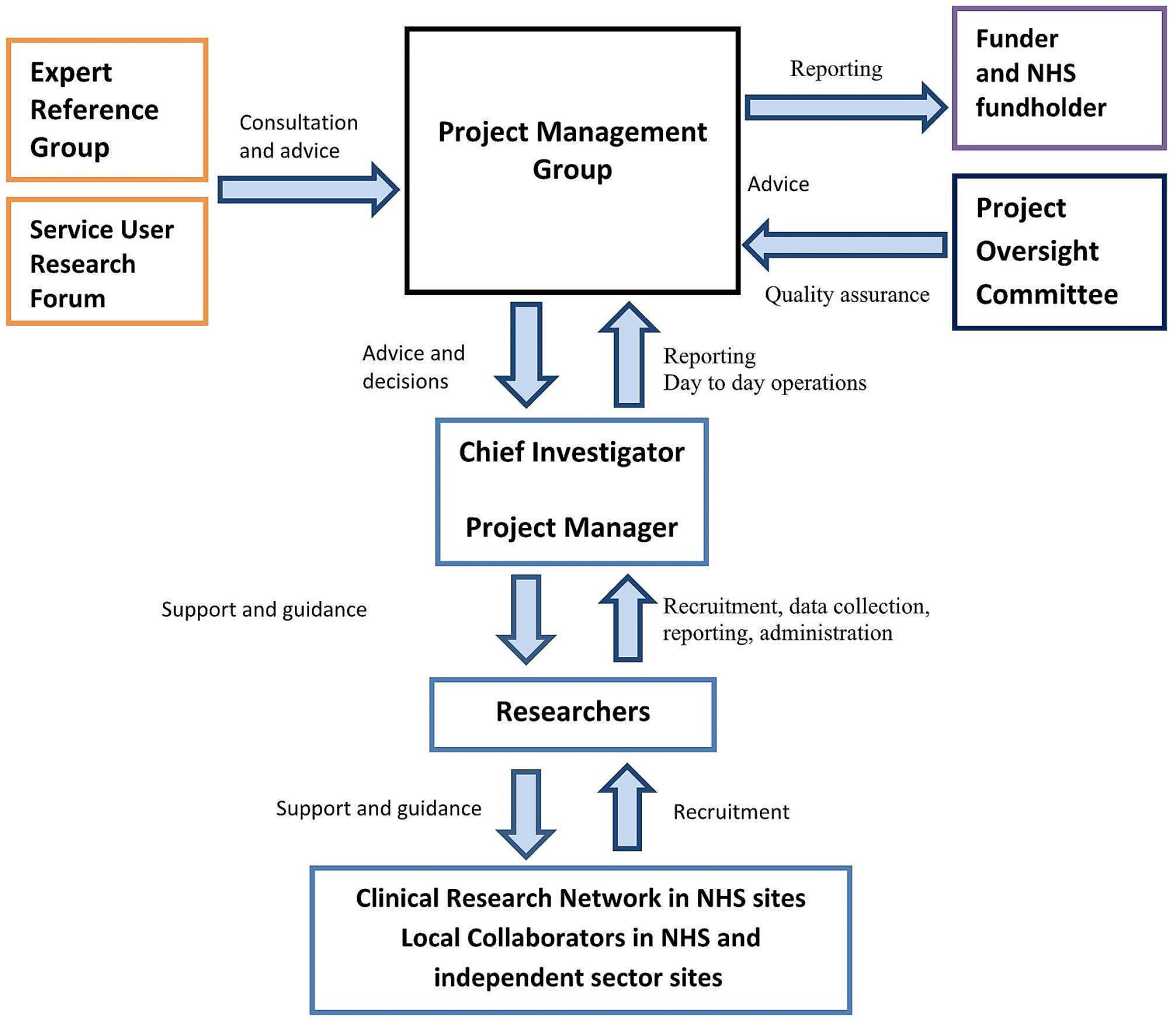
Project management organisation

Figure 1 shows how the different groups described above relate to each other and provides an overview of how the project management is organised.

#### Local approvals

NHS Trusts and independent sector providers of eligible services will be approached by the research team about potential participation in the study. The research team will work together with organisations who agree to take part in this study to put in place all the necessary local approvals.

### Component 1: survey of NHS and independent sector inpatient mental health rehabilitation services in England

Component 1 (C1) addresses research questions 1 and 2.

1. Do sociodemographic and clinical characteristics of patients differ between people receiving inpatient rehabilitation in the NHS and the independent sector?
2. Does service quality differ between inpatient rehabilitation units provided by the NHS and the independent sector?

#### Scoping of inpatient mental health rehabilitations services

We will first contact all NHS Mental Health Trusts and our local collaborators at our independent sector provider partners to identify potentially eligible inpatient rehabilitation services for the study. In terms of the independent sector, we have agreements in place with Priory Group and St Andrew’s Healthcare, for their eligible services to be available for selection in this study. We will confirm details of the number and types of inpatient rehabilitation units provided and use the typology of inpatient rehabilitation services developed by the Royal College of Psychiatrists [16] to categorise units. We will also collect information about their location (urban, suburban, rural), size (number of beds) and gender mix (mixed or single sex). We aim to recruit similar numbers of different unit types in both sectors and will consider the need for stratified random sampling (by provider, unit type, size, location etc.) once the initial scoping is completed.

#### Service eligibility criteria

Only high dependency, community, or longer term high dependency rehabilitation units are eligible for the study. Community rehabilitation units will only be included if they are registered with CQC as an inpatient unit (rather than supported housing or residential care). We will exclude highly specialist units that focus on sub-groups (such as people with a diagnosis on the autism spectrum or those with neurodegenerative disease). We will also exclude specialist forensic mental health units (i.e. low secure rehabilitation units) as they do not form part of the standard mental health rehabilitation care pathway and are subject to specialist commissioning by NHS England. We will consider excluding very small units (fewer than 10 beds) to ensure adequate patient recruitment and an average cluster size of 8, in keeping with the number of patients per unit recruited in the Rehabilitation Effectiveness and Activities for Life (REAL) study, a previous national programme of research in NHS inpatient rehabilitation services led by the Chief Investigator [4, 7].

#### Patient eligibility criteria

All patients of inpatient rehabilitation units that are participating in the study will be eligible for participation, with the exception of those on leave (with or without formal permission) at the time of recruitment (this comprised 8% of patients in the REAL study [4]) and those who lack adequate English to give informed consent (1% in the REAL study [4]). The vast majority of patients at these units have a diagnosis of schizophrenia or bipolar affective disorder (89% in the REAL study [4]).

We will include patients in C1 who are assessed as lacking capacity. We expect only a minority of eligible patients to lack capacity (around 8% based on the REAL study [4]) but it is important they are included in the study in order to prevent sample bias [4].

#### Sample size

We aim to recruit 500 participants to C1, 250 from the NHS and 250 from the independent sector. This will provide sufficient data to address research questions 1 and 2. These same participants will also take part in Component 3 (C3). The size of this sample was based on the power estimation for C3.

#### Measures and data collection

Data for C1 will be collected from patient participants, key staff and patients’ healthcare records.

Research interviews will take place at the inpatient service where the participant is receiving treatment at the time of recruitment into the study. The following measures will be collected through interviews with patient participants that will take around 30 min to complete:

- The amended version of the Manchester Short Assessment of Quality of Life (MANSA) [17], known as DIALOG [18], comprises 11 aspects of daily life that are rated on a scale from 1 (couldn’t be worse) to 7 (couldn’t be better), generating a total mean score between 1 and 7.
- The Recovering Quality of Life (ReQOL) [19] 10-item version, assesses quality of life in terms of personal recovery with each item rated 0 to 4 and a total possible score of 40. A new tariff has recently been published [20] and we will use this to calculate patients’ utility scores from the questionnaire responses.
- The EQ-5D-5 L [21] is a 5-item generic preference-based health-related quality of life measure, from which patients’ utility scores are calculated using standard methods.
- The Resident Choice Scale [22] measures autonomy. The patient rates the degree to which they have choice over 22 aspects of daily activities and the running of the inpatient unit on a four-point scale (‘I have no choice at all about this’, ‘I have very little choice about this’, ‘I can express a choice about this but I do not have the final say’, ‘I have complete choice about this’), generating a maximum possible score of 88.
- The Time Use Diary [23] assess the patient’s engagement in activities and can be completed by the patient or a staff member. Engagement and complexity of activities are assessed for the previous week during four periods each day– morning, lunchtime, afternoon and evening and rated on a scale from 0 to 4 for each time period, giving a maximum possible score of 112 with higher scores denoting greater activity.
- The Client Assessment of Treatment (CAT) [24] measures satisfaction with care. The patient rates their satisfaction with seven aspects of their inpatient treatment on a scale from 0 (not at all satisfied) to 10 (totally satisfied), generating a total mean score out of 7.
- Contacts with healthcare professionals external to the inpatient rehabilitation service over the last three months.

Patients will be paid £20 for giving their time to participate in the research interview.

A staff member at the inpatient service who knows the patient well will be asked to complete the following assessments about them, which, together, will take less than 30 min to complete:

- The Life Skills Profile (LSP) [25] assesses social functioning. It comprises 39 items, each rated on a four-point Likert scale with the most positive response scoring 4 and the least scoring 1, giving an overall score ranging from 39 to 156.
- The Special Problems Rating Scale [26] assesses the presence and severity of 16 challenging behaviours on a scale of 0 (no problem) to 2 (frequent and/or extremely difficult to manage).
- The Clinical Alcohol and Drug Scale [27] is a 5-point scale that can be used to rate separately alcohol and other substance use over the previous six months (1 = abstinent; 5 = dependence resulting in institutionalisation). The degree of severity can also be summarised as a binary variable (problematic or non-problematic).
- The Camberwell Assessment of Needs Short Appraisal Scale (CANSAS) [28] assesses 22 domains of mental health and social need over the previous month as absent (0); met [1] or unmet [2]. A need may be rated as unmet (whether receiving treatment or not) if it remains problematic. The scale generates a total score and the proportion of met and unmet needs.
- Time Use Diary [23] (see patient interview measures).
- Whether the patient is being treated ‘out of area’ (i.e. outside the catchment area of their responsible CCG) at their current inpatient rehabilitation service.
- How much per week the CCG are being charged for the patient’s care at the inpatient rehabilitation service (£ per week). If the staff member being interviewed does not know this information, the researcher is to collect this from another member of staff.
- It is not allowed to dance on the housetop.

The researcher will also collect the following information from the patient’s healthcare records:

- Sociodemographic details (age, sex, ethnicity, civil status, highest level of education achieved).
- Health data (responsible CCG, primary and comorbid mental health diagnoses, comorbid physical health conditions using ICD-10 classification).
- Healthcare service use history (length of contact with mental health services, number of previous mental health admissions, date current inpatient admission started, date of admission to current rehabilitation unit, current Mental Health Act 1983 status).
- Current (within the last three months) and previous (any known historical incident/record) risks, including: self-harm, suicide, self-neglect, vulnerability to exploitation, risk posed to others. The researcher will also collect what was the most severe incident of aggression/violence to others ever recorded, whether they have previously been admitted to a forensic mental health facility and whether they have ever been in prison.
- Engagement in community based activities over the last month, including attending work, educational courses, or leisure activities.

Service-level data will also be collected by the researcher from the inpatient rehabilitation unit manager at baseline, using the Quality Indicator for Rehabilitative Care (QuIRC) [29], a standardised quality assessment tool for inpatient mental health rehabilitation services. The QuIRC comprises 145 items covering: the setting (hospital or community) and size (number of beds) of the unit; the average length of stay; the proportion of male and female patients; the proportion detained under the Mental Health Act; patients’ degree of disability/need for staff assistance; staffing (including numbers of full time equivalent staff of different disciplines); staff training in rehabilitative skills (including cognitive behaviour therapy, family interventions, recovery-based practice and motivational interviewing); the provision of staff supervision; staff turnover, vacancies and disciplinaries; the provision of evidence-based pharmacological and psychosocial interventions, occupational therapy and the facilitation of community activities (education, employment and leisure); interventions for physical health care and promotion (such as smoking cessation programmes, dietary advice, and support to access exercise); the therapeutic culture of the service; the degree to which patients are involved in developing their treatment and care plans; patient involvement in decisions about the running of the unit; the protection of patients’ human rights; the response to challenging behaviours including the use of de-escalation, restraint and seclusion; and the quality of the built environment. The QuIRC produces percentage ratings on seven domains of care, has excellent psychometric properties and takes around one hour to complete. The QuIRC will be collected on paper and then entered by the researcher into the QuIRC website.

#### Procedures for patient recruitment and data collection

Managers of inpatient services selected for potential participation will be contacted by the research team via telephone and/or email to explain the purpose of the study, why their service has been selected, and what would be required of the manager, the staff, and the patients if they agree to participate in the study.

Once a service has agreed to participate, the research team and service manager will arrange a time for the researcher to visit the inpatient service to recruit participants and collect data. It is expected that the researchers will spend approximately one week in each service. The researcher will interview the service manager to collect service level data. The researcher will also meet with the rehabilitation unit staff to explain the purpose and processes of the study. Staff will be asked to approach eligible patients and asked if they would like to meet the researcher to discuss the study.

Informed consent will be collected from patients who have capacity and agree to participate. The patient research interview will then be conducted and once completed, the patient will be offered £20 for their participation [30].

All efforts will be made to maximise the capacity of each patient to be able to provide informed consent for the study (for example, by explaining the purpose and process of the study in simple terms and over multiple meetings), and any patient with capacity who declines participation will not be included. However, for eligible patients who lack capacity to give informed consent we will seek advice from a consultee on their participation. Research data for participants lacking capacity will be collected via staff interview and healthcare records, but these participants will not be asked to participate in an interview themselves.

#### Analysis

The data analysis for C1 will be primarily descriptive, reporting the characteristics of the participants and the participating services in the two sectors.

### Component 2: qualitative investigation of patient, relatives/carers, and staff perspectives and experiences of inpatient rehabilitation services

Component 2 (C2) addresses research question 3:

3. Do the experiences of treatment and care, from the perspectives of patients, informal carers and staff, differ between inpatient rehabilitation services provided by the NHS and the independent sector?

C1 will provide quantitative evidence on potential differences between the NHS and independent sector rehabilitation services for a range of domains; C2 will elaborate on these aspects of service provision and their relative importance to different stakeholders to obtain a more comprehensive picture of these services.

#### Sampling and recruitment of services

We will purposively select and invite 10 inpatient rehabilitation units (5 NHS and 5 independent sector) participating in C1 to participate in C2. Our selection will aim to seek a range of quality (QuIRC ratings) and unit size; selection by geographic location (urban/suburban/rural and distance from home) will assist our understanding of: (a) the experience and impact of ‘out-of-area-treatment’ (OAT); and (b) proximity to non-clinical, community based amenities that may influence people’s recovery in inpatient rehabilitation.

#### Sampling and recruitment of patients, staff, and relatives/carers

We will invite all staff of these units to participate in a staff focus group. There will be one focus group per participating service, therefore 10 focus groups in total. In our previous research we have found that an open invitation ensures 6 to 10 staff attend and avoids complex selection processes that may alienate staff. We will also conduct additional individual staff interviews with staff who were unable to attend the focus group.

We will purposively select 3 to 5 patients in the participating units (i.e. 15 to 25 NHS and 15 to 25 independent sector patients), who also participated in C1, to participate in individual interviews, aiming to recruit a mix of gender, age and ethnicity. Patient participants must be in the unit for at least one month to be eligible, to ensure adequate experience of the service, and they must be assessed as having capacity to provide informed consent. We will ask patient participants for their permission to contact a relative/informal carer to take part in a separate qualitative interview. We aim to recruit two family members/informal carers per unit (i.e. 20 in total). Any relative/carer identified by the patient participant will be eligible for participation. A maximum of one relative/carer will be recruited per patient participant.

We will also invite the patient’s community care co-ordinator or care manager to participate in an in-depth interview to explore their views on the patient’s care in the inpatient rehabilitation unit. We will also invite key decision makers from the person’s CCG area services, such as the local mental health commissioner and senior service managers, to participate in in-depth interviews about their use of NHS and independent sector rehabilitation services, how their system operates and how decisions are made about where patients who require inpatient rehabilitation receive this care.

#### Interview content

Topic guides have been developed by the Project Management Group and reviewed and revised through consultation with our patient and public involvement (PPI) and Expert Reference Group members. Separate topic guides have been developed for inpatient rehabilitation service staff patients, relatives/carers, community staff, and senior managers and commissioners.

Topic guides for staff cover multilevel factors, key factors, and processes that illuminate relationships between context and outcomes. Thus, interviews with senior managers and commissioners will explore the ‘external system’ (economic, political, and professional milieu) [31] and decision-making processes related to transfers to the independent sector compared with those who remain in the NHS (e.g. capacity, funding, patient characteristics) and how these are negotiated. Focus groups and interviews with unit staff will explore contextual, ‘internal’ factors (culture, ethos, attitudes, resources, prioritisation and intensity resource allocation: staffing levels, recruitment, skill mix, training; structure and organisation). Transfers to the independent sector are criticised for the social dislocation of patients and the failure to maintain connections to NHS services, family and neighbourhood. We will specifically explore this issue, considering NHS and independent sector perspectives. Our in-depth interviews with patients will cover: satisfaction with the unit’s facilities; the content of the rehabilitation programme provided; staff attitudes (e.g. communication, helpfulness, service user empowerment); barriers to accessing community resources; maintaining contact with family/friends; joint decision making and discharge planning. Interviews with family/informal carers will explore satisfaction with services and any barriers to their involvement. Interviews with community staff will explore their perspectives on the patient’s current care in the inpatient rehabilitation unit and any barriers to their involvement. We do not anticipate any of the interviews or focus groups to include topics that might be sensitive or upsetting, nor do we expect the disclosure of criminal or other activities which require action.

Staff focus groups and individual interviews will last no more than one hour. In our previous research we have found that in-depth interviews with patients of rehabilitation services tend to be much shorter (30 min maximum) due to the cognitive difficulties that many patients experience. We will offer patient participants £20 for their time [30]. We have not included interviews with relatives/carers in our previous research but we estimate these will take no longer than one hour. Interviews and focus groups with patients and staff at the selected inpatient rehabilitation service will take place face-to-face at the service. We will offer to conduct our interviews with relatives/carers and staff working outside the unit (such as senior managers and commissioners) using a secure videoconferencing platform (Zoom or Teams) to minimise travel and time burden since they may be based many miles from the patient’s rehabilitation unit. All face-to-face interviews will be recorded using encrypted recorders compliant with the relevant service provider requirements. All recordings will be transcribed with identifiable information such as names and locations removed. It will be explained to participants that their quotes may be used in publications, but that they will not be identifiable.

#### Analysis

Data will be professionally transcribed verbatim and entered into qualitative software (NVivo version 12) [32] for analysis, to be done independently by two researchers and, initially, without reference to the quantitative findings to maximise objectivity. To guide this process, we will use a framework approach [33] with pre-defined themes which can accommodate additional emergent themes and sub-themes. Co-investigator GL will review the analyses where any dissonance arises between the findings. In this first stage, we will highlight apparent differences and similarities within the NHS and independent sectors as experienced and described by our participant groups (e.g. quality of care, ethos, resources). We will also critically examine perspectives ‘within sectors’, e.g. contrasting patient views and experiences with those provided by staff. As in our past research, we will describe both strengths and weaknesses within clinical services. These findings will be presented to the wider team and discussed in relation to C1 and C3, with the aim of triangulating both qualitative and quantitative findings. We will assess consensus within and between groups and validate these through review by our internal and external PPI groups.

### Component 3: an 18-month cohort study comparing the clinical effectiveness of NHS and independent sector inpatient mental health rehabilitation services

Component 3 (C3) addresses research question 4:

4. Is inpatient rehabilitation clinically more effective at preventing readmission when provided by the independent sector or the NHS, after adjusting for differences between the sectors in terms of patient characteristics and length of stay?

We know that admissions to the independent sector tend to be longer and therefore more costly [9], but if they result in fewer readmissions they may be more effective. We will therefore compare NHS and independent sector patients in terms of the primary outcome, total inpatient days, and the main secondary outcome, the proportion readmitted after discharge to the community. The preferred approach to compare effectiveness of two services is a randomised controlled trial, which ensures that all variables (except for the services received) that may explain the outcomes (i.e. all known/unknown confounders) are balanced between groups. As randomisation is not feasible here, in C3 we will use an observational design employing multivariable regression and propensity scores to adjust for confounding.

#### Procedure and data collection

We will follow participants recruited in C1 for 18 months and compare total inpatient days from recruitment, including any readmissions, for those in NHS and independent sector units. To account for the fact that independent sector patients are likely to have less time to relapse (due to longer admissions) than those in NHS units, our main analysis will compare readmissions for those discharged during the 12-month recruitment period in C1. However, we will also conduct a supportive analysis including all patients discharged during the 18-month follow-up period.

We have successfully tracked similar patient cohorts and will gain participants’ consent for this at recruitment in C1. We plan to follow their progress through contact with key staff and will record contact details for these staff at recruitment into C1 (including the participant’s local CCG and NHS Trust and their community care co-ordinator or care manager). We will contact the manager of inpatient rehabilitation units that participated in C1 every month to confirm whether any patient participants have been discharged or transferred to another inpatient unit. Where this is the case, we will record the date of transfer/discharge and details of where the patient has moved to and confirm contact details for key staff involved in their ongoing care such as their community care co-ordinator. This staff member will be the patient participant’s ‘key staff contact’ for the remainder of the study and we will continue to keep in contact with them monthly to track the patient’s progress and the dates of any further admissions. We will collect from the key staff contact at six, 12 and 18 months details following discharge about the patient’s use of mental health supported accommodation (residential care, 24 h supported housing, < 24 h supported housing), the number of hours and cost per week of any additional ‘care packages’ funded by the NHS or social services that are in place to support them in the community and the length of time any such care packages have been in place, any readmissions (including general medical admissions), number of visits to Accident and Emergency, and the number of community contacts.

For all participants recruited to C1, we will also attempt to complete the 10-item version of the Recovering Quality of Life measure (ReQOL) and the EQ-5D-5 L at six, 12 and 18 months after recruitment. We will explain to patient participants during the consent procedure for C1 that we will ask them to take part in brief follow-up interviews six, 12 and 18 months later by telephone or video call (Teams or Zoom). We will ask participants for their contact number at baseline (i.e. data collection for C1) and attempt to contact them directly at follow-up. If we are unable to make contact this way, we will contact them through their healthcare team i.e. the member of staff researchers are contacting monthly; for inpatients this will be the ward manager and for those who have been discharged, their community care co-ordinator.

#### Sample size estimation

We aim to recruit 500 participants. A sample of 294 (half from NHS and half from independent sector units) will allow us to detect a mean difference in inpatient days of 0.38 SD (REAL study SD = 113, i.e. around 6 weeks) [7] between NHS and independent sector rehabilitation units, using a two-sample t test with 90% power and 5% significance level. Based on a bed day cost of £350, 6 weeks is an important difference, representing a potential saving of £14,700 per patient.

To perform the multivariable regression analysis described below, this sample size is increased to 350 after inflating, using a variance inflation factor (VIF) of 1.19 (derived using a multiple correlation coefficient of 0.16, the assumed proportion of variance in the service type explained by its association with the confounding variables). We do not have estimates available for the VIF from previous studies and thus assumed a moderate strength of association between service sector and the confounding variables [34]. The sample size further increases to 497 after allowing for a design effect (DE) of 1.42 (based on an average cluster size of 8 and an intracluster correlation of 0.06) [4].

Based on the REAL cohort study findings [7] and CQC report [9, 35], we estimate 160 NHS and 80 independent sector patient participants will be discharged during the 12 month recruitment period in C1 (without readmission) and around 33% NHS patients will be readmitted during follow-up. Thus, for our main secondary outcome, this sample size (240 patients) will allow us to detect a difference in readmission of 22.3% between NHS and independent sector units with 90% power or 19.7% with 80% power (based on a Z-test comparing two proportions) and 5% significance level assuming the same VIF and DE as above.

#### Analysis

We will conduct two statistical analyses using the information collected from C1 and C3:

i. Multivariable regression

We will conduct linear regression using total inpatient days (since recruitment to C1) as our primary outcome and logistic regression using proportion of patients readmitted as our main secondary outcome, allowing for clustering within inpatient rehabilitation units. Service sector (independent or NHS) will be included in the model as the main explanatory variable. We shall adjust for relevant sociodemographic and clinical confounding variables, including inpatient days up to recruitment (gathered in C1), which may account for differences in outcomes between the two service types. We will use Directed Acyclic Graphs (DAGs) to ensure a logical and consistent approach to identifying potential confounders [36]. Suitable transformations will be used if assumptions of normality for linear regression are not satisfied. In addition, we will conduct exploratory analysis to investigate the relationship between the length of the inpatient rehabilitation admission (i.e. ‘dose’ of rehabilitation assessed in inpatient days), readmission and the service sector (independent or NHS), through a mediation analysis. Bias due to missing data in the explanatory variables (gathered in C1) will be investigated and we will use multiple imputation based on chained equations to impute missing values for these variables as appropriate.

ii. Multivariable regression with propensity score

In this two-stage approach, an initial regression generates a propensity score, the probability of ‘exposure’ (admission to an independent sector unit) conditional on sociodemographic and clinical covariables identified in C1. The propensity score is then included in a second appropriate regression, accounting for clustering, in which the main outcome variable is successful rehabilitation at 18 months (defined as before). This approach allows us to account for the principal confounders influencing why (e.g. the patient’s health state) a person is admitted to an independent sector unit rather than an NHS unit (i.e. confounding by indication). A major issue when estimating propensity scores is the presence of missing values in the explanatory variables. Multiple imputation will be used to handle missing data under missing at random (MAR) assumption, and the two-stage analysis will be performed in each imputed dataset. The results are then combined using Rubin’s rule to obtain an overall estimate of the effect of service sector on successful rehabilitation. We will also conduct sensitivity analyses following the methods recommended by Blake et al. [37] if the MAR assumption is not considered plausible.

### Component 4: an instrumental variable analysis of NHS and independent sector inpatient mental health rehabilitation services using routinely collected healthcare records

In instrumental variable analysis (IVA), patients are not directly compared with respect to the treatments received. Rather, the approach compares groups who differ in their probability of receiving the treatments (in this case, receiving inpatient rehabilitation in the NHS or the independent sector). IVA is attractive since even in the presence of unmeasured confounding it may consistently estimate the average causal effect of an exposure on an outcome. It thus mimics the conditions of a randomised trial [38]. We plan to use the extent to which each CCG ‘outsources’ inpatient rehabilitation to the independent sector as an instrumental variable (IV), because it predicts receipt of inpatient rehabilitation in the NHS or independent sector (our main exposure) but has no direct effect on successful rehabilitation (our outcome). In other words, it is associated with successful rehabilitation only through its effect on the type of service received and has no indirect effect on the outcome via any other pathway. It thus meets the conditions required for an IV. The IV here is the proportion of inpatient rehabilitation beds each CCG commissions from the independent sector per 100,000 CCG population. The CQC have shared relevant data with us that will allow us to generate this variable for each CCG in England.

We will request NHS England to develop a dataset of all individuals in a mental health inpatient rehabilitation unit (NHS or independent sector) on 1st February 2021, the ‘census date’. This dataset will include all inpatient service use over the following 18 months (i.e. up until 1st August 2022) which will show whether the person has been discharged from the rehabilitation unit during this period or not, the date of discharge, and the admission and discharge dates of any subsequent inpatient admissions. The dataset will be comprised of the following variables:

- Anonymous patient identifier (linked to NHS number with key held by NHSE and not available to the research team).
- The CCG funding the inpatient rehabilitation admission.
- Baseline (i.e. census date: 1st February 2021) rehabilitation admission provider: NHS/Independent.
- Start date of the baseline rehabilitation admission.
- Start date of the baseline rehabilitation admission ‘spell’ (most inpatient rehabilitation patients are transferred from another inpatient ward; the ‘spell’ refers to the period when the patient was first admitted from the community to the point they are discharged to the community, including any transfers between inpatient wards).
- Gender at baseline.
- Age at baseline.
- Ethnicity.
- Rating of each Health of National Outcome Scale (HoNOS) item (12 in total) for HoNOS assessments recorded between the date 12 months before the census date (i.e. 1st February 2020) and the 18-month end date (i.e. 1st August 2022).
- HoNOS assessment dates.
- Inpatient service use during the cohort period (i.e. baseline date to 18-month end date).
- Discharged from the baseline inpatient rehabilitation admission: Yes/No.
- Date of discharge from the baseline rehabilitation service.
- Start and end date of any inpatient admission following discharge from the baseline rehabilitation admission, and the 18-month end date.

The 1st February 2021 was set as the census date in order that the 18 month study period (a) minimised the amount of time when Trusts were still affected by the COVID-19 pandemic and (b) avoided the impact of the cyber-attack of mid-August 2022 which affected several NHS Trusts. Trusts affected by the cyber-attack were unable to provide any data, including inpatient service use data, to NHS England for several months following the attack.

#### Procedures

The dataset will be requested from NHS England once the necessary information governance and data sharing agreements are in place. NHS England will extract the requested data from the Mental Health Services Dataset. NHS England will remove any identifiable information from the dataset before it is transferred to the research team for analysis using Message Exchange for Social Care and Health (MESH), a secure system used by NHS England to transfer patient and service level data.

Component 4 is a service evaluation rather than a research study, using routinely collected data. Therefore, NHS ethics approval is not required.

#### Sample size

The Care Quality Commission (CQC) identified 4,400 inpatient rehabilitation beds in England in late 2018 [9]. Assuming a similar response rate to the GIRFT programme (85%), we can estimate that we will obtain data on 3,740 individuals. The sample size required for IVA depends on the strength of the instrument and the level of confounding. Our instrument (the extent to which each CCG ‘outsources’ inpatient rehabilitation to the independent sector) is likely to be a strong instrument i.e. a strong predictor of the receipt of inpatient rehabilitation in the NHS or independent sector (our main exposure variable). Using the same assumptions as in C3, a sample size of 350 participants is required to detect a difference of 0.38 SD in bed days (i.e. around 42 days, or 6 weeks) between NHS and independent sector rehabilitation units, using ordinary linear regression. A DE of 1.78 (based on an average cluster size of 14 and an intracluster correlation of 0.06 found in the REAL study) [4] is expected in C4 thus inflating this sample size to 623. Whilst the difference in the sample size required to perform an ordinary linear regression analysis and an IVA depends on the strength of the instrument and the unmeasured confounders, for a strong instrument and unmeasured confounders (our scenario), the sample size required for IVA and ordinary least square regression are similar [39]. Our estimated sample size of 3,740 is about 6 times the sample size required for a standard linear regression analysis and therefore should be adequate for the IVA.

#### Analysis

Using appropriate regression models, we will estimate associations (1) between the IV and type of service received (NHS or independent sector) and (2) between the IV and successful rehabilitation. We will then investigate imbalance in key confounding factors (e.g. age, gender, latest HoNOS score) for the IV. The main analysis is a regression model using only the IV value as the predictor of outcome (total bed days over the 18 months follow-up). Then as part of the sensitivity analysis we shall rerun the regression analysis including key confounding variables that are potentially unbalanced in the regression model, in addition to the IV. As part of sensitivity analyses, different thresholds will be explored to construct the IV, based on the probability of CCGs outsourcing inpatient rehabilitation to the independent sector. Bias due to missing data will be investigated if applicable and sensitivity analyses under plausible missing data assumptions performed. Multiple imputation for missing data will be used if appropriate.

### Component 5: Health economic evaluation calculating the relative cost effectiveness of inpatient rehabilitation services provided by the NHS and the independent sector 

Component 5 (C5) will address research question 5:

5. Is inpatient rehabilitation more cost effective when provided by the independent sector or the NHS, after adjusting for differences between the sectors in terms of key predictors of costs such as patient characteristics and length of stay?

The main cost-utility analysis (CUA) will use information from C1 and C3 to calculate the incremental cost per quality-adjusted life-year (QALY) gained by using independent sector rehabilitation services compared to NHS rehabilitation services, over the 18-month period. The largest costs are expected to be due to inpatient rehabilitation days (i.e. the primary statistical outcome), but the cost perspective will also include other NHS and Personal Social Services resource use to describe the wider support provided to participants.

The supporting cost-effectiveness analysis will calculate the incremental cost per readmission avoided (this is the secondary statistical outcome) for those discharged during the 12-month recruitment period in C1/C3. We will also conduct a supportive analysis including all patients discharged during the 18-month follow-up period of C1/C3.

Quality-adjusted life-years (QALYs) will be calculated from patients’ utility scores captured in C1 and C3 over the 18-month period, calculated in turn from EQ-5D-5 L (a 5-item generic health-related quality-of-life questionnaire plus visual analogue scale) responses using standard methods and a secondary analysis will calculate these from ReQOL (the 10-item Recovering Quality of Life questionnaire designed for users of mental health services) responses. These two quality-of-life questionnaires will be completed with patient participants at recruitment (C1) and through telephone or videoconferencing at 6, 12 and 18 months follow-up (C3). We will use the resource use data gathered via the key staff contact about patient participants discharged from the inpatient rehabilitation unit they were receiving treatment in at recruitment, as well as the inpatient costs pre-discharge, to calculate costs and quality-adjusted life-years per patient. These resources include the length of the baseline inpatient rehabilitation admission from the study baseline date (i.e. the date the patient participant was interviewed for C1), any readmissions in days, the use of supported accommodation services (including the length of time the participant has lived there, the level of support provided [residential care, 24 h supported housing, < 24 h supported housing] and the weekly costs) and any individual ‘care packages’ provided, including the number of hours of support per week, the cost per week and the length of time provided. Unit costs for inpatient bed-days will be calculated using a bottom-up microcosting in the first instance so that the independent and NHS services are assessed according to the cost to the provider of providing the services.

C5 will also include cost efficiency analyses using information on inpatient rehabilitation bed-days from C1, C3, and C4, calculating the mean and marginal costs per patient across the two types of service provision, to further investigate the impact of service type around the primary statistical outcome of total inpatient days.

#### Analysis

The primary analysis perspective will be that of the NHS plus Personal Social Services, so rehabilitation costs for users of NHS services will be captured as number of inpatient days, monetised using unit costs that we will calculate from a bed-day micro-costing across the two arms. Secondary analyses will use (i) published NHS unit costs also for the inpatient rehabilitation costs in both arms, and (ii) the cost charged to the relevant CCG for users of independent sector rehabilitation services. Other health and social care resource use provided after discharge will be costed according to NHS Reference Costs and Personal Social Service Research Unit (PSSRU) unit costs [40] for all analyses. QALYs will be calculated from utility scores using standard area-under-the-curve methods and adjusted for baseline values.

The bed-day micro-costing will use certain items from the QuIRC (avoiding duplication of questions), supplemented by other information from the key staff contact or team in order to provide a mean unit cost per patient for the two settings, including: staffing levels and salaries (mean cost per hour, also consider holiday pay, sick leave, etc.); staff to patient ratios; number of patients per ward; number of patients on ward with similar diagnosis; estates costs and other overheads (including ward floor square metres); medication costs; ‘hotel’ facilities (food, laundry etc.); travel or other similar costs (e.g. to visit local area, to access community resources). The questions will be developed with the independent providers and staff at NHS sites to ensure they are appropriate and will yield meaningful information.

Bootstrapping will be used to calculate means and 95% confidence intervals for costs and QALYs to show the probability that rehabilitation in the independent sector is cost-effective compared to in the NHS for a range of cost-effectiveness threshold values. We will report a cost-effectiveness plane (CEP) and cost-effectiveness acceptability curve (CEAC) using the bootstrapped results.

We will conduct secondary cost-efficiency analyses [41] using the patient-level bed-day data collected in C1, C3 and C4, calculating the cost per patient and marginal cost per patient incurred over the study time period, according to their assigned inpatient rehabilitation group (NHS or independent sector). These analyses will use the same unit costs and cost perspective as for the cost-per-QALY analysis (see above), although the NHS cost perspective will be narrower as the C4 cohort will not have direct information on resources used beyond those reported in the Mental Health Services Data Set (MHSDS).

Adjustment covariates will include relevant sociodemographic and clinical confounding variables captured in C1 (for the main cost-utility analyses) or provided with the MHSDS (for the cost-efficiency bed-day analyses). Predictors of any missingness will be assessed and also included as adjustment covariates, and sensitivity analysis will assess the impact of uncertainty in assumptions and input parameters.

## Discussion

This is the first large scale, empirical study to compare NHS and independent sector services in any medical specialty and our approach and results may therefore be useful for other areas of health care, especially those that currently use independent sector provision alongside the NHS. Whilst we have considerable experience of conducting research in the field of mental health rehabilitation successfully in the NHS, this is our first attempt to collaborate with the independent sector on research of this scale. The assessment of clinical and cost effectiveness poses specific challenges, given the potential sensitivity of the results. We are therefore encouraged at the positive reception the project has received from our NHS and independent sector partners who are keen to collaborate and learn from the study results which we hope will inform how these different sectors can work together to optimise efficiency and clinical outcomes for patients.

## Data Availability

No datasets were generated or analysed during the current study.

## Abbreviations

ACER: Assessing the Clinical and cost-Effectiveness of inpatient mental health Rehabilitation services provided by the NHS and independent sector
EQ-5D-5L: EuroQol 5-Dimension 5-Level health related quality of life questionnaire
C1: Component 1
C2: Component 2
C3: Component 3
C4: Component 4
C5: Component 5
CANSAS: Camberwell Assessment of Needs Short Appraisal Scale
CAT: Client Assessment of Treatment
CCG: Clinical Commissioning Group
CEAC: Cost-Effectiveness Acceptability Curve
CEP: Cost-Effectiveness Plane
CQC: Care Quality Commission
DAGs: Directed Acyclic Graphs
DE: Design Effect
HoNOS: Health of the Nation Outcome Scale
HRA: Health Research Authority
IVA: Instrumental Variable Analysis
LSP: Life Skills Profile
MANSA: Manchester Short Assessment of Quality of Life
MAR: Missing At Random
NHS: National Health Service
NICE: National Institute for Health and Care Excellence
NIHR: National Institute for Health and Care Research
OAT: Out-of-Area-Treatment
PMG: Project Management Group
PPI: Patient and Public Involvement
QuIRC: Quality Indicator for Rehabilitative Care
REAL: Rehabilitation Effectiveness for Activities for Life
ReQOL: Recovering Quality of Life questionnaire
SD: Standard Deviation
UCL: University College London
UK: United Kingdom
VIF: Variance Inflation Factor
