# Correction: Assessing the clinical and costeffectiveness of inpatient mental health rehabilitation services provided by the NHS and independent sector (ACER): protocol

**DOI:** 10.1186/s12888-024-05859-0

**Published:** 2024-05-29

**Authors:** Helen Killaspy, Christian Dalton-Locke, Caroline S Clarke, Gerard Leavey, Artemis Igoumenou, Maurice Arbuthnott, Katherine Barrett, Rumana Omar

**Affiliations:** 1 Division of Psychiatry, University College London, 6th Floor, Maple House, 149 Tottenham Court Road, London, W1T 7NF UK; 2 Camden and Islington NHS Foundation Trust, St Pancras Hospital, 4 St Pancras Way, London, NW1 0PE UK; 3 Research Department of Primary Care and Population Health, University College London, UCL Medical School, Upper 3rd Floor, Royal Free Campus, Rowland Hill Street, London, NW3 2PF UK; 4 Bamford Centre for Mental Health & Wellbeing, Ulster University, Cromore Road, Coleraine, County, Londonderry, BT52 1SA UK; 5 Barnet, Enfield and Haringey Mental Health NHS Trust, St. Ann’s Hospital, St Ann’s Rd, London, N15 3TH UK; 6 North London Service User Research Forum, Division of Psychiatry, University College London, 6th Floor, Maple House, 149 Tottenham Court Road, London, W1T 7NF London UK; 7 Department of Statistical Science, University College London, 1-19 Torrington Place, London, WC1E 7HB UK

**Correction: BMC Psychiatry (2024) 24:104** 10.1186/s12888-024-05524-6

Following the publication of the original article [1], the authors identified that the bed day cost in the sub-heading Sample size estimation was incorrect. The correct bed day cost is given below.

The incorrect bed day is: £3503.

The correct bed day is: £350.

The original article [1] has been corrected.
